# An ultra-sensitive suboptimal protospacer adjacent motif enhanced rolling circle amplification assay based on CRISPR/Cas12a for detection of miR-183

**DOI:** 10.3389/fbioe.2024.1444908

**Published:** 2024-09-18

**Authors:** Zhiquan Lu, Shijing Wang, Ping Li, Huasheng Yang, Sanyang Han, Shaochong Zhang, Lan Ma

**Affiliations:** ^1^ Precision Medicine and Healthcare Research Center, Tsinghua-Berkeley Shenzhen Institute (TBSI), Tsinghua Shenzhen International Graduate School, University Town of Shenzhen, Shenzhen, China; ^2^ Institute of Biopharmaceutical and Health Engineering, Tsinghua Shenzhen International Graduate School, Tsinghua University, University Town of Shenzhen, Shenzhen, China; ^3^ Shenzhen Eye Hospital, Jinan University, Shenzhen Eye Institute, Shenzhen, China; ^4^ State Key Laboratory of Ophthalmology, Zhongshan Ophthalmic Center, Guangdong Provincial Key Laboratory of Ophthalmology and Visual Science, Sun Yat-sen University, Guangzhou, China; ^5^ Institute of Biomedical Health Technology and Engineering, Shenzhen Bay Laboratory, Shenzhen, China

**Keywords:** diabetic retinopathy, CRISPR/Cas12a, microRNA, rolling circle amplification, fluorescence

## Abstract

**Introduction:**

MicroRNAs (miRNAs) have been recognized as promising diagnostic biomarkers for Diabetic Retinopathy (DR) due to their notable upregulation in individuals with the condition. However, the development of highly sensitive miRNAs assays for the rapid diagnosis of DR in clinical settings remains a challenging task.

**Methods:**

In this study, we introduce an enhanced CRISPR/Cas12a assay, leveraging suboptimal PAM (sPAM)-mediated Cas12a trans-cleavage in conjunction with rolling circle amplification (RCA). sPAM was found to perform better than canonical PAM (cPAM) in the detection of Cas12a-mediated ssDNA detection at low concentrations and was used instead of canonical PAM (cPAM) to mediate the detection. The parameters of reactions have also been optimized.

**Results and discussion:**

In comparison with cPAM, sPAM has higher sensitivity in the detection of ssDNA at concentrations lower than 10 pM by Cas12a. By replacing cPAM with sPAM in the padlock template of RCA, ultra-high sensitivity for miR-183 detection is achieved, with a detection limit of 0.40 aM. within 25 min and a linear range spanning from 1 aM. to 1 pM. Our assay also exhibits exceptional specificity in detecting miR-183 from other miRNAs. Furthermore, the applicability of our assay for the sensitive detection of miR-183 in clinical serum samples is also validated. This study introduces a groundbreaking assay with excellent performance through a simple modification, which not only addresses existing diagnostic challenges, but also opens exciting new avenues for clinical diagnosis in the realm of DR.

## 1 Introduction

Diabetic retinopathy (DR), an ocular microvascular complication of diabetes occurring in the retina, is the most common causes of blindness in developed nations (Zhang et al., 2012). Most DR patients are at risk of vision loss within 5–10 years without treatment (Akbari Kordkheyli et al., 2021). Early diagnosis of DR is vital for the control and treatment of DR progression. At present, ocular fundus inspection was the most common screening method of DR, but limited by media opacities and the lack of gold standards for lesion detection (Bek, 2016). Therefore, a rapid and sensitive detection methods is indeed needed for the DR early diagnosis.

MiRNAs are a class of small (∼22 nt) non-coding RNAs that regulates gene expression by targeting mRNA for cleavage or translational repression, and play a vital role in regulation of gene expression in various biological processes (Bartel, 2004; Turchinovich et al., 2012). Aberrant expression of specific miRNAs can be found in a variety of diseases, suggesting that miRNAs are potential and promising disease biomarkers (Fu et al., 2011; Mendell and Olson, 2012; Siasos et al., 2020; Wang et al., 2016). For instance, miR-21 was found upregulated in several types of cancers and miR-155 was found upregulated in breast cancer (Cheng, 2015; Eichelser et al., 2013; Le et al., 2012; Nguyen et al., 2021). Several miRNAs have also been found to be aberrantly expressed in the development of DR (Dähmcke et al., 2023; Wang et al., 2023; Zhao and Pan, 2023). MiR-183 was reported to be significantly over-expressed in DR, and meanwhile, inhibition of miR-183 expression could repress the development of DR, indicating that miR-183 may serve as biomarker for DR diagnosis (Wu et al., 2012; Zhang et al., 2019).

Since the abnormal expression of miRNAs is closely related to the development of diseases, they are often used as biomarkers. However, due to the low expression, small size, and high sequence similarity of miRNAs, sensitive detection of miRNAs is still challenging (Cissell et al., 2007; Deng et al., 2017). Rolling circle amplification (RCA) is a simple and versatile isothermal amplification strategy for short DNAs or RNAs. In a typical RCA process, a ssDNA padlock template (PT) is ligated into circular structure with the assistance of enzymes and target DNA or RNA, and further generates long ssDNA or ssRNA with hundreds of tandem repeats (Ali et al., 2014; Jonstrup et al., 2006; Murakami et al., 2009). Due to its isothermal nature and high customizability, RCA has become powerful tool in bioassays for a variety of targets including RNA, DNA, proteins and pathogens (Gao et al., 2019; Tang et al., 2018; Tian et al., 2019; Zhang et al., 2018; Zhu et al., 2023).

In recent years, the clustered regularly interspaced short palindromic repeats (CRISPR)/Cas system has been widely used for pathogen and disease DNA and RNA detection and received a lot of attention since its discovery as a powerful gene editing tool (Barrangou et al., 2007; Marraffini and Sontheimer, 2008; Su et al., 2024). Among the many CRISPR/Cas systems, Cas12a (Cpf1) is widely used for the construction of high-performance DNA/RNA detection platforms due to its RNA-guided sequence-specific recognition and endonuclease activity. The recognition of Cas12a is based on the guidance of crRNA with a T-rich protospacer adjacent motif (PAM), which lead to specific cleavage of target DNA, known as cis-cleavage, and non-specifically cleavage of existing ssDNA, known as trans-cleavage (Chen et al., 2018; Qian et al., 2019; Zetsche et al., 2015). Taking advantage of the trans-cleavage effect of Cas12a on reporter ssDNA, a series of highly sensitive nucleic acid detection systems have been developed, most of which rely on cumbersome primer design or experimental manipulation, as well as advanced processing and characterization devices (Cui et al., 2021; Hang et al., 2022; Huang et al., 2023; Ke et al., 2022; Peng et al., 2023; Shen et al., 2023; Zhao et al., 2023). Recently, a one-pot assay based on suboptimal PAM (sPAM) has been developed for the one-pot detection of RNA. The sPAM is a PAM with a modified base sequence that has a lower reaction rate towards the same target compared to the canonical PAM (cPAM), hence named suboptimal PAM. In the one-pot reaction of recombinase polymerase amplification (RPA) and CRISPR/Cas12a trans-cleavage, the slower kinetics of sPAM-mediated trans-cleavage leads to reduced template consumption, consequently facilitating the amplification of target, which in turn improves the sensitivity of detection (Lu et al., 2022).

Herein, we propose a novel method based on sPAM-mediated CRISPR/Cas12a reaction for the ultra-sensitive detection of miR-183 for DR diagnosis. The proposed method simply introduces sPAM into the RCA and subsequent CRISPR/Cas12a trans-cleavage processes, and can achieve attomolar sensitivity in the detection of miR-183. In this study, we designed a miR-183-specific PT with a combination of sPAM and spacer, which produces long ssDNA with tandem repeating sequences for the subsequent recognition of Cas12a/crRNA complex. A 5-nt-long F-Q-labeled ssDNA was employed as probe for the characterization of trans-cleavage event. The RCA-sPAM-Cas12a (RSC) assay exhibited an ultra-high sensitivity of detecting miR-183 with a detection limit of 0.4 aM in a wide linear range of 1 aM to 1 pM under optimal conditions. Finally, this method showed a sensitive detection rate to distinguish DR positive patients from all human serum samples, indicating its potential in early clinical diagnosis of DR.

## 2 Materials and methods

### 2.1 Clinical sample sources

In this study, all clinical samples were obtained with informed consent from patients, and the study was approved by Zhongshan Ophthalmic Center, Sun Yat-sen University. The samples collected and used in this work were serum samples from untreated DR-negative and DR-positive patients with a diagnosis of fundus photography.

### 2.2 Reagents and apparatus

T4 RNA Ligase 2 (T4 Rnl2) and Phi29 polymerase were purchased from New England BioLabs, LbCas12a and F-Q probe were purchased from Bio-lifesci (Guangzhou, China), Gel-red nucleic acid gel stain, dNTPs mix and Rnase inhibitor were purchased from Beyotime Biotech (Jiangsu, China), 20 bp DNA Ladder was purchased from Takara Bio (Beijing, China), 30% Acr-bis (29:1) was purchased from Servicebio (Wuhan, China), other chemical reagents were purchased from Macklin (Shanghai, China). All the primers were synthesized by Sangon (Shanghai, China) and the sequences were shown in Supplementary Table S1. Quantification of extracted RNA was performed by a NanoDrop One UV-Vis microspectrophotometer (Thermal Fisher). The electrophoresis results were visualized on a WB-9413B gel imaging analyzer (Liuyi biotech, Beijing). All the nucleic acid reactions were conducted in a ProFlex™ 3 × 32 thermal cycler (Thermal Fisher), the fluorescence spectra were recorded by a qTOWER^3^ real-time fluorescence quantitative PCR instrument (Analytik Jena AG).

### 2.3 RCA procedure for miR-183

The RCA process consists of two steps: ligation and polymerization. The ligation step was carried out in a volume of 20 µL containing 10 µL of target miR-183 with different concentration, 500 nM padlock template (PT), 20 U Rnase inhibitor, 4 U T4 Rnl2, 2 µL of 10×T4 Rnl2 reaction buffer (500 mM Tris-HCl (pH 7.5 at 25°C), 20 mM MgCl_2_, 10 mM dithiothreitol (DTT)), 4 mM Adenosine triphosphate (ATP)) and diethyl pyrocarbonate (DEPC)-treated water. The reaction mixture was incubated at 25°C for 1 h.

In the polymerization step, 5 µL of the ligation product was added into the 35 µL polymerization reaction mixture containing 4 µL of 10 × Phi29 DNA polymerase reaction buffer (500 mM Tris-HCl (pH 7.5 at 25°C), 100 mM MgCl_2_, 100 mM (NH_4_)_2_SO_4_, 40 mM DTT), 4 µg of recombinant albumin, and 1.5 µL of dNTPs (25 mM for each of dATP, dTTP, dCTP and dGTP), 10 U Phi29 DNA polymerase (10 U/µL) and DEPC-treated water. The mixture was incubated at 30°C for 2 h and then terminated at 65°C for 10 min.

### 2.4 CRISPR/Cas12a reaction

The CRISPR/Cas12a reaction was performed in a volume of 60 µL containing 10 µL of target ssDNA or RCA products, 6 µL of 10 × NEBuffer 2.1 (10 mM Tris-HCl (pH 7.9 at 25°C), 50 mM NaCl, 10 mM MgCl_2_, 100 μg/mL bovine serum albumin (BSA)), 60 U of Rnase inhibitor, 500 nM of ssDNA probe, 150 nM crRNA, 100 nM LbCas12a and DEPC-treated H_2_O. The mixture was incubated at 37°C for 25 min and then terminated at 65°C for 10 min. The CRISPR/Cas12a cleavage step was carried out in a real-time fluorescence quantitative PCR instrument and its fluorescence emission intensity at 535 nm under 485 nm excitation was recorded every 30 seconds.

### 2.5 Gel electrophoresis

To analyse the RCA products, non-denaturing poly-acrylamide gel electrophoresis (8% PAGE) was performed in 1×TBE buffer. The 8% PAGE was conducted at 120 V constant voltage for 30 min and the gel was stained by Gel-red for 15 min.

## 3 Results

### 3.1 Working principle of miR-183 detection

**FIGURE 1:**
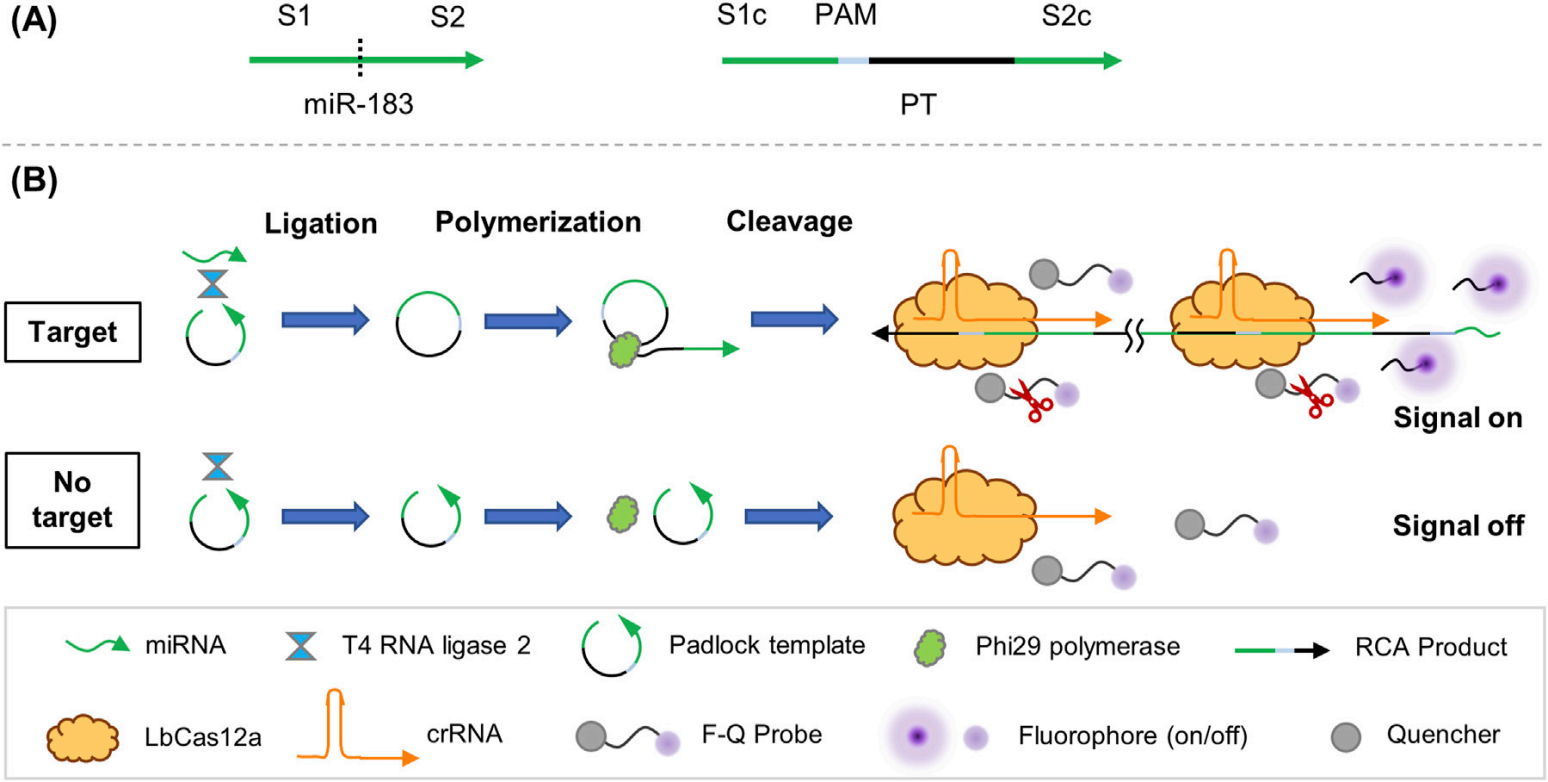
Working principle of RSC system. **(A)** Design of PT. **(B)** Schematic illustration of RSC in detection.

The design of PT and schematic illustration of miR-183 detection is depicted in Figure 1. The miR-183 serves as spacer in the CRISPR/Cas12a reaction and is split into two segments: S1 and S2, with the complementary sequences S1c and S2c located at two ends of PT to form a padlock. PAM is located near the end of the split spacer to non-specific recognition of unlinked template by crRNA-Cas12a complex. In the RSC assay, sPAM CTTA is used to mediate CRISPR/Cas12a reaction instead of cPAM TTTA.

The RSC assay consists of three steps: ligation, polymerization and cleavage. In the presence of target miR-183, both ends of PT join and form a loop structure under the ligation of T4 Rnl2. The ligated loop PT works as template in the polymerization step and produce long ssDNA with repetitive sequence in the presence of Phi29. After the RCA process, the product of RCA is transferred to the CRISPR/Cas12a detecting system, in which the repeated sPAM and subsequent spacer of RCA product is recognized by crRNA-Cas12a complex and triggers trans-cleavage of F-Q probe, resulting in accumulated fluorescence signal. On the contrary, no RCA or subsequent trans-cleavage occurs in the absence of miR-183, and therefore no fluorescence signal would be generated.

### 3.2 Characterization of RCA

**FIGURE 2:**
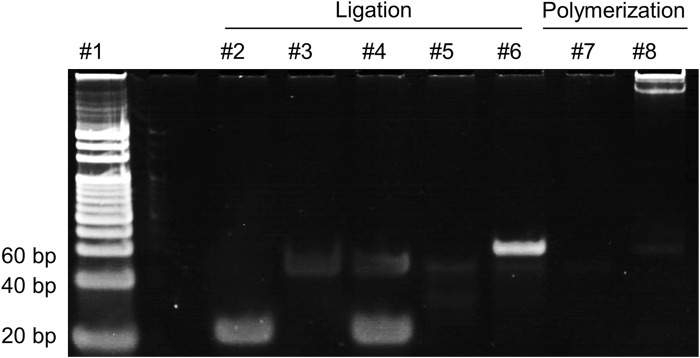
8% PAGE analysis of the products in the RCA reaction. lane 1: 20 bp DNA ladder; lane 2: miR-183 (400 nM); lane 3: PT (500 nM); lane 4: miR-183 + PT; lane 5: PT + T4 Rnl2; lane 6: miR-183 + PT + T4 Rnl2; lane 7: products of lane 5 + Phi29; lane 8: products of lane 6 + Phi29.

The products of ligation and polymerization in the RCA process were subjected to 8% PAGE and the results are presented in Figure 2. Comparing lane 6 with lane 4 and lane 5, a distinct band in lane 6 can be observed, indicating that the ligation was successfully conducted in the presence of miR-183, while no such band of ligation was observed in the group without T4 Rnl2 (lane 4) or miR-183 (lane 5). In the polymerization step, a band with large molecular weight can be observed in lane 8, indicating that the RCA was successfully performed and generated a large amount of long ssDNAs, while no pattern of amplification was observed without miR-183 (lane 7). The results above demonstrate successful RCA activated by miR-183.

### 3.3 Investigation of sPAM-mediated CRISPR/Cas12a reaction

**FIGURE 3:**
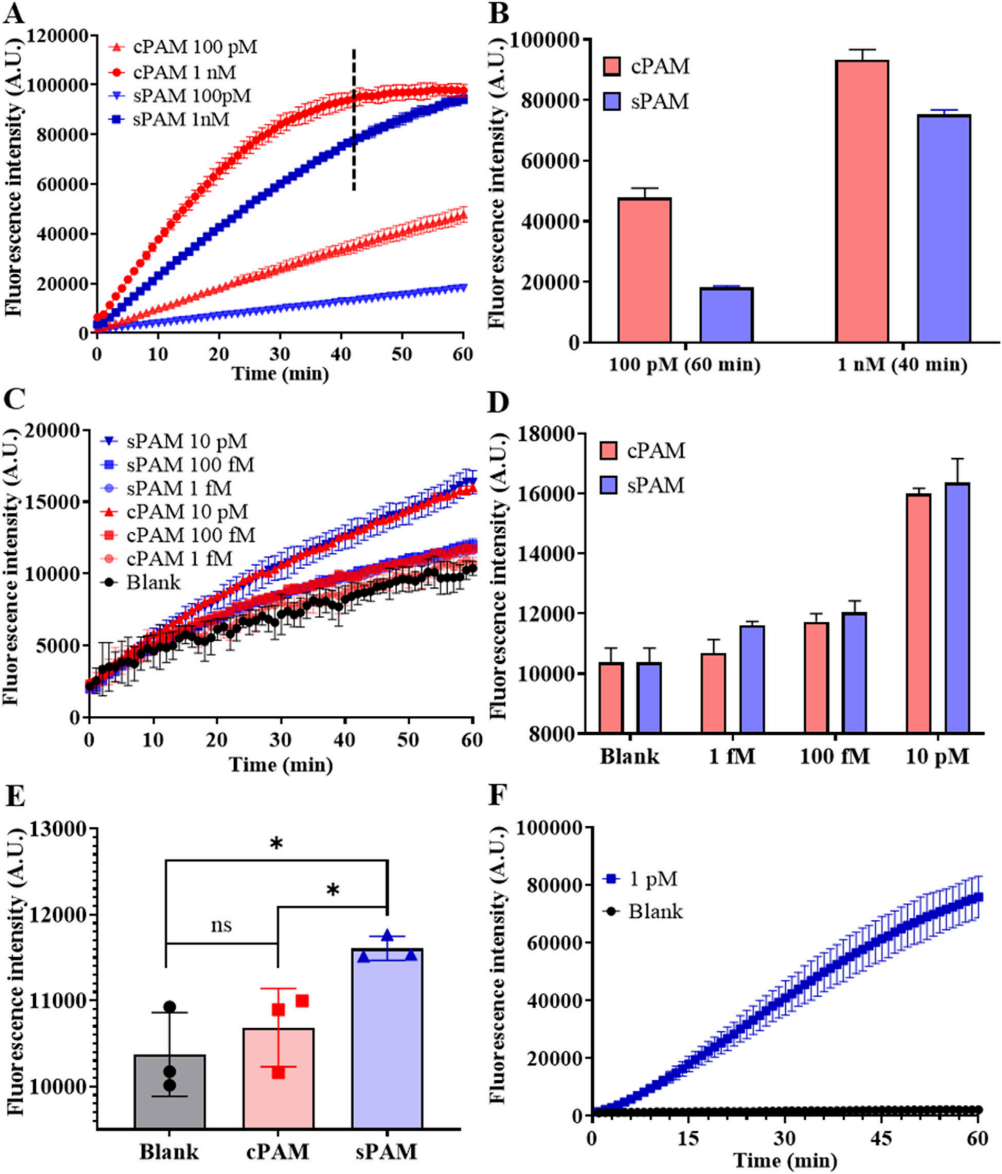
Investigation of sPAM-mediated CRISPR/Cas12a detection and RSC detection. **(A)** Real-time fluorescence intensity of cPAM- and s-PAM mediated CRISPR/Cas12a reaction of ssDNA at high concentrations of 100 pM and 1 nM. **(B)** Fluorescence intensity at the end of 60-min reaction for 100 pM and 40-min reaction for 1 nM. **(C)** Real-time fluorescence intensity of cPAM- and sPAM-mediated CRISPR/Cas12a reaction of ssDNA at low concentrations. **(D)** Fluorescence intensity at the end of 60-min ssDNA detection. **(E)** Comparison of cPAM- and sPAM-mediated 1 fM ssDNA detection. **(F)** Detection of miR-183 at a concentration of 1 pM using RSC. (Mean ± s.d., n = 3 technical replicates. Variance was calculated using student’s t-method, * represents *p* < 0.05.).

To explore the feasibility of sPAM in an individual CRISPR/Cas12a ssDNA detection reaction, two target ssDNAs with different PAMs was designed. The two target ssDNAs have a spacer complementary to the same crRNA, and different PAMs, which are sPAM CTTA and cPAM TTTA, respectively. Fluorescence intensity of CRISPR/Cas12a trans-cleavage in a 60 µL reaction was employed to characterize the performance of detection and the results are shown in Figure 3. As depicted in Figures 3A,B, both cPAM- and sPAM-mediated CRISPR/Cas12a assay showed their ability in detection of target ssDNA at concentrations larger than 100 pM. Moreover, the cPAM-mediated reaction was much more efficient than that of sPAM, and the cPAM-mediated detection at 1 nM was completed in 40 min.

However, this rule is gradually reversed as the concentration of the detection target decreases. As depicted in Figures 3C,D, when the target concentration was reduced to 10 pM or lower, the mean values of sPAM group were larger than that of cPAM group, in contrast. The results in Figure 3E further demonstrates that when the concentration of target was reduced to as low as 1 fM, cPAM failed to distinguish between the blank and experimental groups, while sPAM was still capable of distinguishing them, with a significant difference from cPAM. Therefore, it can be concluded that cPAM is more advantageous than sPAM in the detection of ssDNA at high concentrations, but when the target concentration decreases to 10 pM or lower, sPAM performs better.

In addition, the detection of non-PAM containing template was also performed to investigate the influence of PAM, and non-PAM did not perform as well as sPAM and cPAM due to its low recognition and cleavage efficiency, as shown in Supplementary Figure S2.

Feasibility of the whole RSC system for miR-183 detection was also investigated and the result is shown in Figure 3F. As depicted in Figure 3F, miR-183 triggered RCA and subsequent trans-cleavage of Cas12a, leading to increased fluorescence signal, while no increase of fluorescence intensity was observed in the blank control, indicating the feasibility of RSC in miR-183 detection.

### 3.4 Optimization of experimental conditions

**FIGURE 4:**
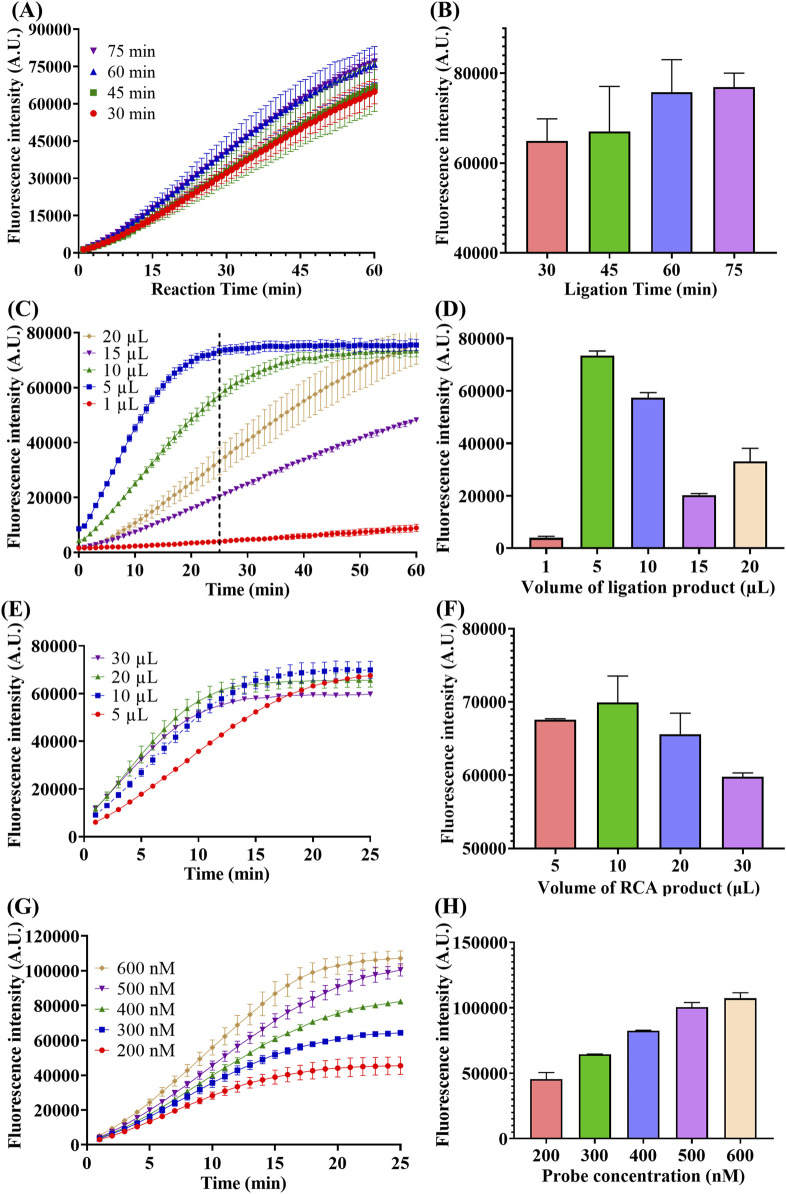
Optimization of ligation time, ligation product volume, RCA product volume and probe concentration. **(A)** Real-time fluorescence intensity of miR-183 (1 pM) detection with different time of ligation. **(B)** Fluorescence intensity at the end of 60-min reaction. **(C)** Real-time fluorescence intensity of miR-183 (1 pM) detection with different ligation product volume, the dotted line represents the 25th minute of the reaction. **(D)** Fluorescence intensity at the end of 25-min reaction. **(E)** Real-time fluorescence intensity of miR-183 (1 pM) detection with different RCA product volume. **(F)** Fluorescence intensity at end of 25-min reaction. **(G)** Real-time fluorescence intensity of miR-183 (1 pM) detection with different concentration of F-Q probe. **(H)** Fluorescence intensity at end of 25-min reaction. (Mean ± s.d., n = 3 technical replicates).

To achieve best performance of RSC method, a series of experiments were conducted to optimize the reaction parameters of the method. Owing to the fact that the multi-step reaction of this system involves too many parameters and it is time-consuming and laborious to optimize all of them, some of the reaction parameters followed the manufacturer’s recommendations or the widely used parameters. Therefore, the optimization of the reaction conditions was focused on the ligation reaction and the influence of the ions introduced in each step was also investigated, with the results shown in Figure 4.

Firstly, the reaction time of ligation was optimized and the result is shown in Figures 4A,B. As depicted in Figure 4A, the fluorescence intensities increased with time in all four groups and there was a positive correlation between fluorescence generating rate and ligation time. This positive correlation can be more intuitively observed from the comparison of the end-point fluorescence intensities in Figure 4B. Moreover, the increase in end-point fluorescence intensity became slower when the connection time exceeded 60 min, and thus 60 min was chosen as the optimal time of ligation.

Secondly, considering the influence of the substantial ions brought by the buffer of each step on the enzyme activities of the subsequent reactions, the volume of products of each step in the next reaction was optimized. As depicted in Figures 4C,D, the addition volume of ligation product had a significant influence on the subsequent polymerization and trans-cleavage reaction, with a 5 µL of addition exhibiting the best performance. It can also be noted from Figure 4C that the fluorescence intensity reached the maximum value within 25 min when 5 µL of ligation product was added. Therefore, 5 µL was selected as the optimal volume of ligation product, and meanwhile, 25 min was chosen as the optimal trans-cleavage time under this condition.

Similarly, the volume of RCA product was also optimized and 10 µL was selected as optimal volume as shown in Figures 4E,F.

Finally, concentration of F-Q probe was optimized to improve the fluorescence generation efficiency. As depicted in Figures 4G,H, the fluorescence intensity was positively correlated with probe concentration and the increase became slower when the concentration was above 500 nM. Therefore, 500 nM was selected as optimal concentration of F-Q probe.

### 3.5 Sensitivity

**FIGURE 5:**
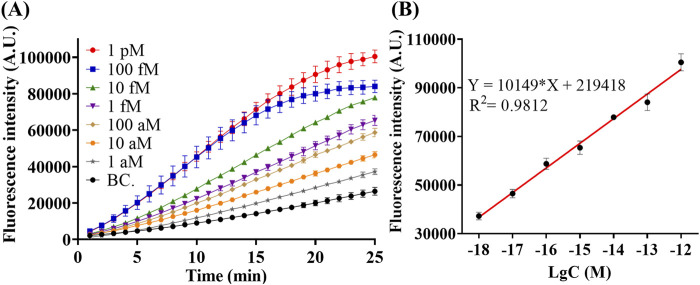
Detection of miR-183 with different concentration under optimal condition. **(A)** Real-time Fluorescence intensity of detection. **(B)** Fluorescence intensity–concentration linear relationship at the end of 25-min reaction. (Mean ± s.d., n = 3 technical replicates for miR-183 positive groups and 13 for blank controls).

Under optimal condition, quantitative detection of miR-183 was performed. As shown in Figure 5A, the fluorescence intensity in the CRISPR/Cas12a trans-cleavage increases along with the increase of miR-183 concentration from 1 aM to 1 pM. Results in Figure 5B further indicates a good linear relationship between fluorescence intensity and concentration of miR-183. The correlation equation was calculated as I = 10,149*lgC + 219,418, with *R* ^2^ = 0.9812, and the limit of detection (LOD) was calculated to be 0.4 aM (IUPAC definition). The RSC exhibited ultra-high sensitivity under optimal conditions.

### 3.6 Specificity

**FIGURE 6:**
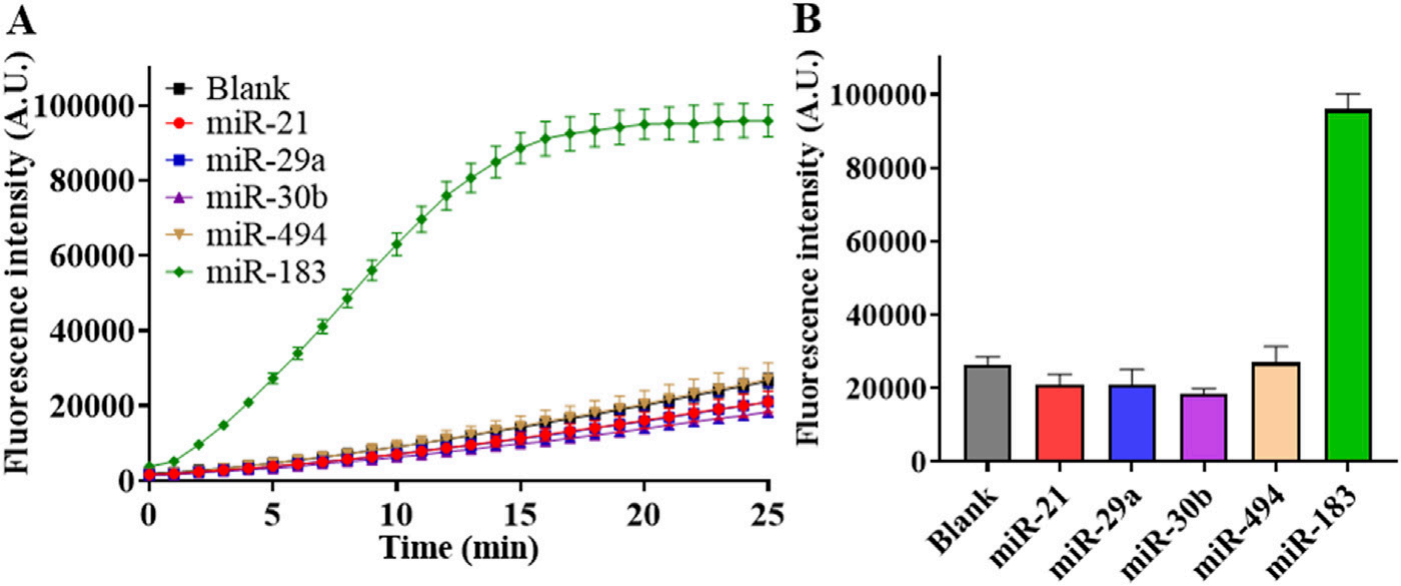
Specificity test of RSC. **(A)** Real-time fluorescence intensity of detection on different miRNAs at concentration of 1 nM. **(B)** Fluorescence intensity at the end of 25-min reaction with different miRNAs. (Mean ± s.d., n = 3 technical replicates for miRNAs and 13 for blank controls).

To evaluate the specificity performance of RSC, we detected different miRNAs at a concentration of 1 nM and recorded their fluorescence intensities. The results in Figure 6A showed that fluorescence intensity of miR-183 detection was significantly higher than groups of other miRNAs, which were very low and close to those of the blank controls (Supplementary Figure S1). In summary, the results in Figure 6 showed that RSC has good specificity in discriminating miR-183 against other miRNAs.

### 3.7 Clinical sample detection

The capability of RSC system in detecting miR-183 in clinical samples was tested, too. The collected serum samples were pre-treated by centrifugation at 15,000 rpm for 15 min to obtain supernatant. The total RNAs were then extracted and quantified by NanoDrop One. Finally, a total amount of 750 ng of extracted RNA for each sample was loaded into the RSC system for the detection of miR-183.

**FIGURE 7:**
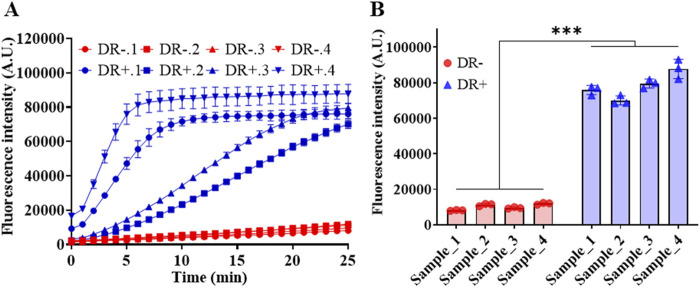
Detection of miR-183 in abstracted total RNA of serum samples. **(A)** Real-time fluorescence intensity. **(B)** Fluorescence intensity at the end of 25-min reaction. (Mean ± s.d., n = 3 technical replicates.

Figure 7 demonstrated the results of miR-183 detection in serum samples of DR -negative and -positive individuals. As depicted in Figures 7A, B, it is evident that miR-183 expression in DR-positive patients is significantly higher than that in DR-negative individuals, and the result is consistent with the diagnosis of fundus photography in the hospital. The high miR-183 expression in DR-positive cases is also consistent with the findings in the literature (Wu et al., 2012). Notably, there is a big difference in the expression of miR-183 among DR-positive patients, according to the real-time fluorescence intensity in Figure 7A, which may be attributed to the different DR progression profiles of patients.

## 4 Conclusion and discussion

DR is a major cause of vision loss in humans, and its early diagnosis is of great significance in controlling and treating the disease. Currently, prevention of DR focuses on regular screening that relies on conventional fundus photography. MiRNA, as a promising biomarker that has been widely reported in the development of DR, there is no miRNA assay for the diagnosis of DR. In this work, we selected miR-183, a potential biomarker for DR and designed a quantitative detection method for it. It is a fluorescence assay that combines RCA and CRISPR/Cas12a trans-cleavage, and enhances the sensitivity of Cas12a by modifying the PAM base sequence. Under optimal conditions, this assay provides an ultra-low LOD of 0.4 aM and a wide linear range of 1 aM–1 pM, and allows for distinguishing DR-positive from DR-negative individuals by detecting the extracted RNA from clinical serum samples.

To date, there are many approaches to detect miRNAs using CRISPR/Cas12a, and most of them are in combination with appropriate amplification methods or complex reaction process and characterization methods. Despite the extremely high rate, the exponential amplification strategy usually requires complex primer design, as well as higher requirements on the experimental environment and operation in order to reduce the risk of false positives. Meanwhile, exponential amplification techniques such as RPA and LAMP are usually combined with reverse transcription to realize miRNA detection, which requires additional primer design and may have specificity issues. The complexity of characterization also limits their further applications in clinical diagnostics. Fluorescence characterization, due to its simplicity and accuracy, has a large number of well-established characterization devices and is more accessible for clinical applications. However, the sensitivity of miRNA fluorescence assays using CRISPR/Cas12a were generally at the femtomolar level, and only a few achieved a sensitivity below 100 aM.

In this method, by simply switching the cPAM bases to sPAM, a great enhancement in sensitivity can be obtained in the detection of ssDNA at low concentrations. The sensitivity of sPAM-based Cas12a detection of ssDNA was measured to be as low as femtomolar level in this paper, enabling more lenient conditions on amplification and broader the application of CRISPR/Cas12a-based assays. Although the linear amplification rate of RCA is not as high as that of exponential amplification, its customizability is highly compatible with sPAM-mediated CRISPR/Cas12a. With an amplification capacity of nearly 1,000-fold, the incorporation of RCA is sufficient to further increase the sensitivity of CRISPR/Cas12a to an attomolar level.

In addition, although previous studies have demonstrated that PAM is not necessary for the detection of ssDNA, this work suggests that modifications to the PAM sequence still have a large impact on the detection of ssDNA, and thus still warrants more in-depth studies to further elucidate the function of PAM in Cas12a-based assays.

In conclusion, we have successfully developed an RCA and sPAM-mediated CRISPR/Cas12a assay for miR-183 detection with an LOD of 0.4 aM and wide linear range of 1 aM to 1 pM. It is simple and ultra-sensitive, and also the first application of sPAM in a stepwise CRISPR/Cas12a detection of miRNA. It also showed a potential application prospect in detection of serum samples of DR patients. This real-time quantitative detection of miR-183 by RSC system may contribute to the diagnosis and more detailed evaluation of DR in the future. Furthermore, the RSC assay is a universal platform that can be applied in the detection of nucleic acid biomarkers for numerous other diseases.

It is important to note that only few miRNA studies are conducted on human DR patients, and most of them are rat-based, so more reliable miRNA biomarkers for human are needed to be discovered in the future.

## Data Availability

The original contributions presented in the study are included in the article/Supplementary Material, further inquiries can be directed to the corresponding authors.
