# Supplementary figures and images for: An ultra-sensitive suboptimal protospacer adjacent motif enhanced rolling circle amplification assay based on CRISPR/Cas12a for detection of miR-183
Source: Front Bioeng Biotechnol. 2024 Sep 18;12:1444908. doi: 10.3389/fbioe.2024.1444908 (PMC11445046; doi:10.3389/fbioe.2024.1444908)

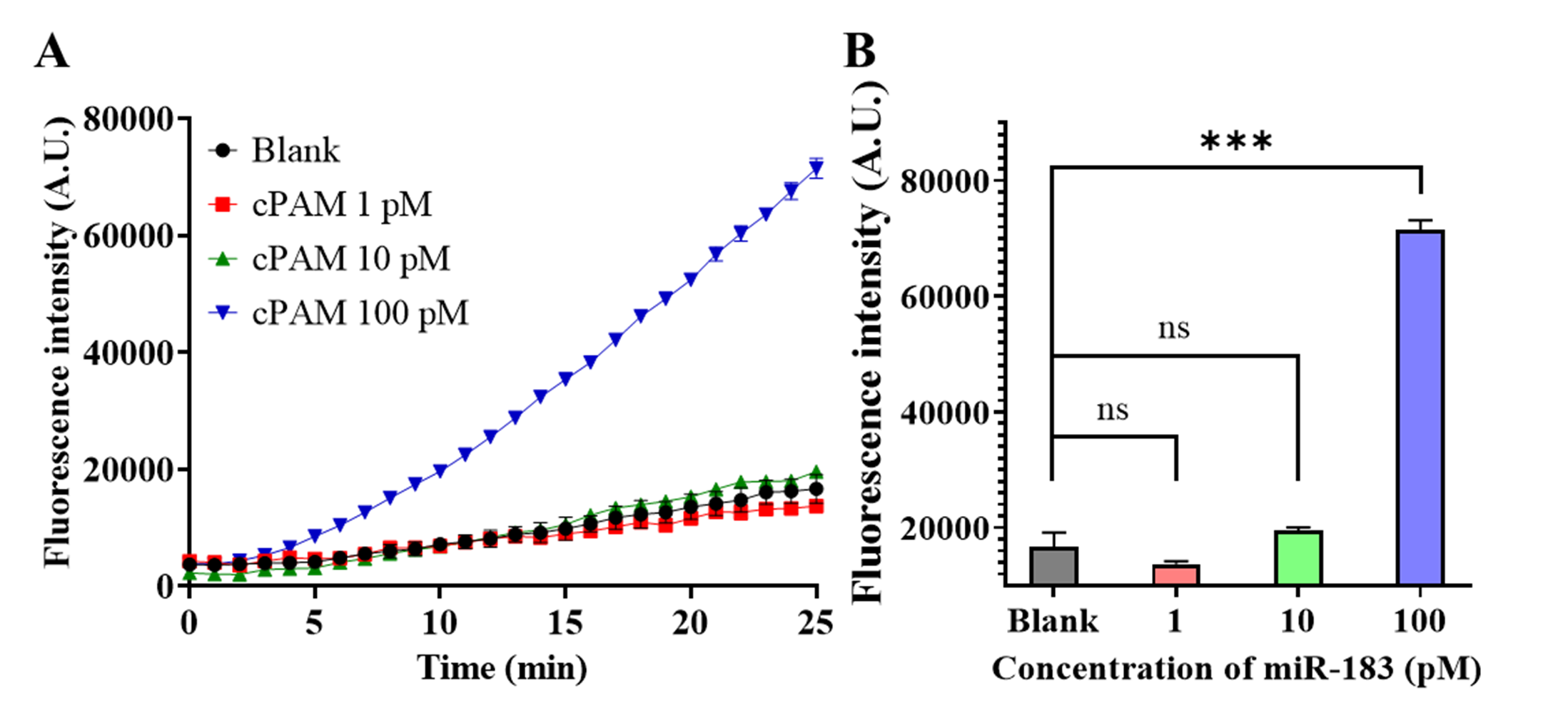

Supplement: Supplementary file 1 [file Image3.TIF]

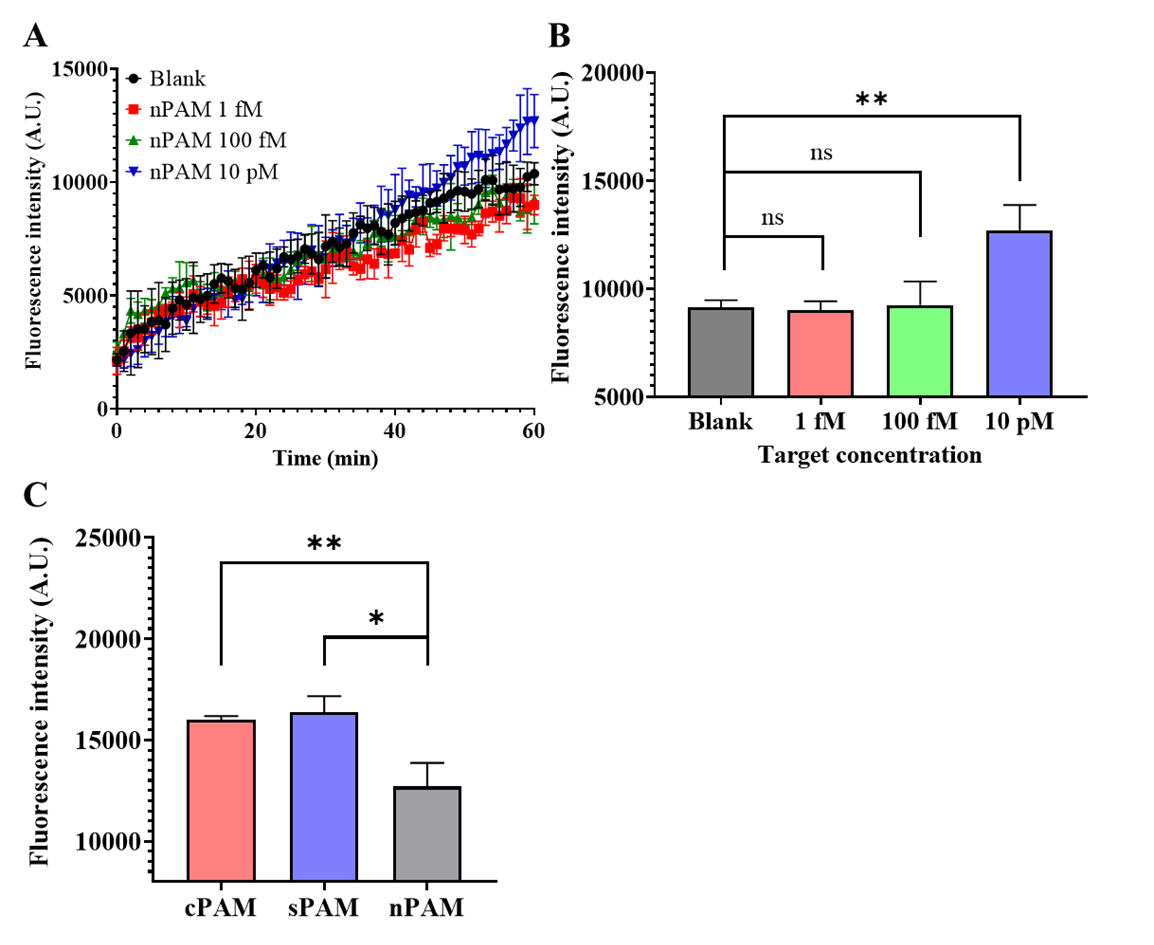

Supplement: Supplementary file 2 [file Image2.TIF]

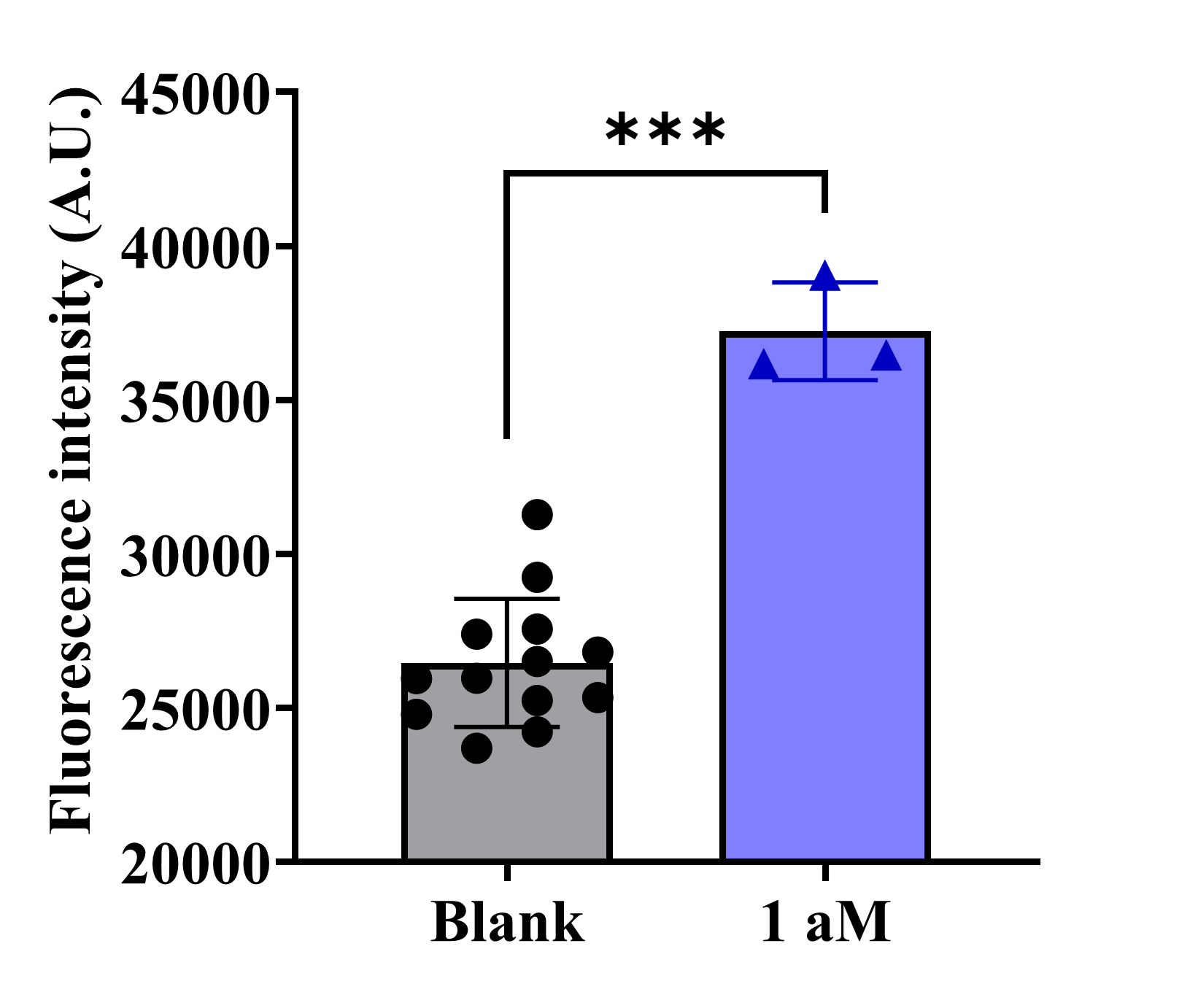

Supplement: Supplementary file 3 [file Image1.TIF]
