# Supplementary material for: An ultra-sensitive suboptimal protospacer adjacent motif enhanced rolling circle amplification assay based on CRISPR/Cas12a for detection of miR-183
Source: Front Bioeng Biotechnol. 2024 Sep 18;12:1444908. doi: 10.3389/fbioe.2024.1444908 (PMC11445046; doi:10.3389/fbioe.2024.1444908)
Supplement: Supplementary file 4 [file DataSheet1.docx]

Supplementary Material

# Nucleic acid sequence involved in this work

**Table S1.** The nucleic acid sequences involved in this work

| Name | Sequence (5’ to 3’) |
| --- | --- |
| ssDNA cPAM | TTGTCTTCCTATGGCACTGGTAGAATTCAC**TAAA**CTGGTTGGTCACTATCACGG |
| ssDNA sPAM | TTGTCTTCCTATGGCACTGGTAGAATTCAC**TAAG**CTGGTTGGTCACTATCACGG |
| ssDNA nPAM | TTGTCTTCCTATGGCACTGGTAGAATTCAC**CGAT**CTGGTTGGTCACTATCACGG |
| miR-183 | UAUGGCACUGGUAGAAUUCACU |
| PT | Phosphate-ACCAGTGCCATAGGAAGACAACCGTGATAGTGACCAACCAG**CTTA**GTGAATT*rCrU* |
| crRNA | UAAUUUCUACUAAGUGUAGAUGUGAAUUCU ACCAGUGCCAUA |
| F-Q probe | 6-FAM-TTTTT-BHQ |
| miR-21 | UAGCUUAUCAGACUGAUGUUGA |
| miR-29a | ACUGAUUUCUU UUGGUGUUCAG |
| miR-30b | UGUAAACAUCCUACACUCAGCU |
| miR-494 | AGGUUGUCCGUGUUGUCUUCUCU |

**Note:** The underlined sequences are hybridization regions for miR-183. In the padlock template, the bold sequences are PAM. The italic nucleotides with letter ‘r’ indicate ribonucleotides.

# Detection of blank control and calculation of LOD

**Supplementary Figure 1.** Comparison of 13 individual experiments of blank control with miR-183 at concentration of 1 aM under optimal conditions. (Mean ± s.d. Variance was calculated using student's t-method, *** represents p<0.001.)

**Calculation of limit of detection.** In the calculation of LOD, the blank samples were tested independently 13 times, and the LOD was calculated according to the IUPAC definition based on equation $I_{L}=x_{b}+k\times s_{b}$, where $I_{L}$ , $x_{b}$ and $s_{b}$represent the LOD, average value and standard variation of samples, respectively. $k=3$ allows a confidence level of 99.86%. The obtained LOD is 0.4 aM.

# The comparison of RSC method with other RCA/Cas12a-based assays for miRNA detection

**Table S2.** The comparison of RSC method with other RCA/Cas12a-based assays for miRNA detection

| Target | Detection strategy | Method of Detection | LOD | Reference |
| --- | --- | --- | --- | --- |
| miR-155 | Cyclic RCA | SERS | 70.2 aM | (He et al., 2017) |
| miR-155 | T-ERCA/Cas12a | Fluorescence | 0.31 fM | (Zhou et al., 2023) |
| Let-7a | RCA + aptamer | Fluorescence | 67.3 fM | (Tang et al., 2018) |
| Let-7a | RCA + LAMP | Fluorescence | 10 aM | (Tian et al., 2019) |
| Let-7a | CHA + Cas12a | Fluorescence | 81.96 fM | (Chen et al., 2022) |
| miR-21 | RCA + Cas12a | Fluorescence | 34.7 fM | (Zhang et al., 2020) |
| miR-21 | RCA + Cas12a | SWV | 0.83 aM | (Qing et al., 2021) |
| miR-183 | RCA + sPAM + Cas12a | Fluorescence | 0.4 aM | This work |

# P-values for each group in ssDNA detection

**Table S3.** The P-values obtained by paired t-test

| Paired t-test | P-value | Significance |
| --- | --- | --- |
| Blank vs. 1 fM cPAM | 0.4623 | NS |
| Blank vs. 1 fM sPAM | 0.0134 | * |
| 1 fM c PAM vs. 1 fM sPAM | 0.0283 | * |
| Blank vs. 100 fM cPAM | 0.0133 | * |
| Blank vs. 100 fM sPAM | 0.0099 | * |
| 100 fM cPAM vs. 100 fM sPAM | 0.4898 | NS |
| Blank vs. 10 pM cPAM | <0.0001 | **** |
| Blank vs. 10 pM sPAM | 0.0004 | *** |
| 10 pM cPAM vs. 10 pM sPAM | 0.4837 | NS |

# Detection of scramble ssDNA


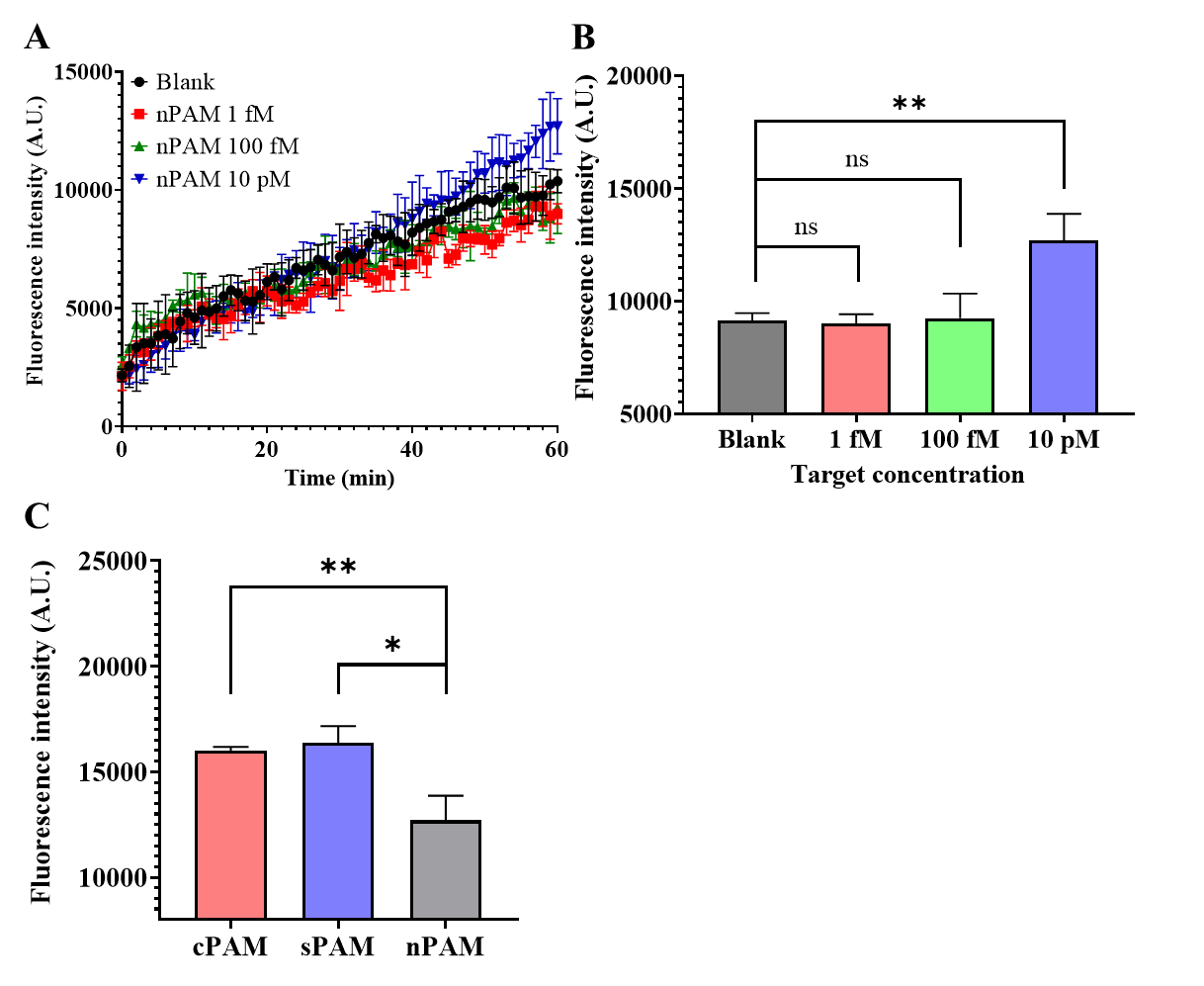


**Supplementary Figure 2.** Detection of scramble ssDNA template by Cas12a. A. Real-time fluorescence of different concentration. B. Comparison of end-point fluorescence intensity for each group after a 60-minute detection. C. Comparison of end-point fluorescence intensity among cPAM, sPAM and nPAM at a concentration of 10 pM. (Mean ± s.d. Variance was calculated using student's t-method, * represents p<0.05, ** represents p<0.01.)

To study the influence of different PAMs, a scramble ssDNA with a non-PAM, denoted as nPAM, was detected. For nPAM template, Cas12a is barely responsive to it when the concentration was below 100 fM. The nPAM template could be distinguished from the blank control when its concentration exceeded 10 pM, indicating the independence of PAM in ssDNA detection. However, the fluorescence intensity was still lower than that of the sPAM and cPAM groups at this point, indicating a lower recognition and cleavage efficiency of nPAM.

# cPAM-mediated MiR-183 Detection


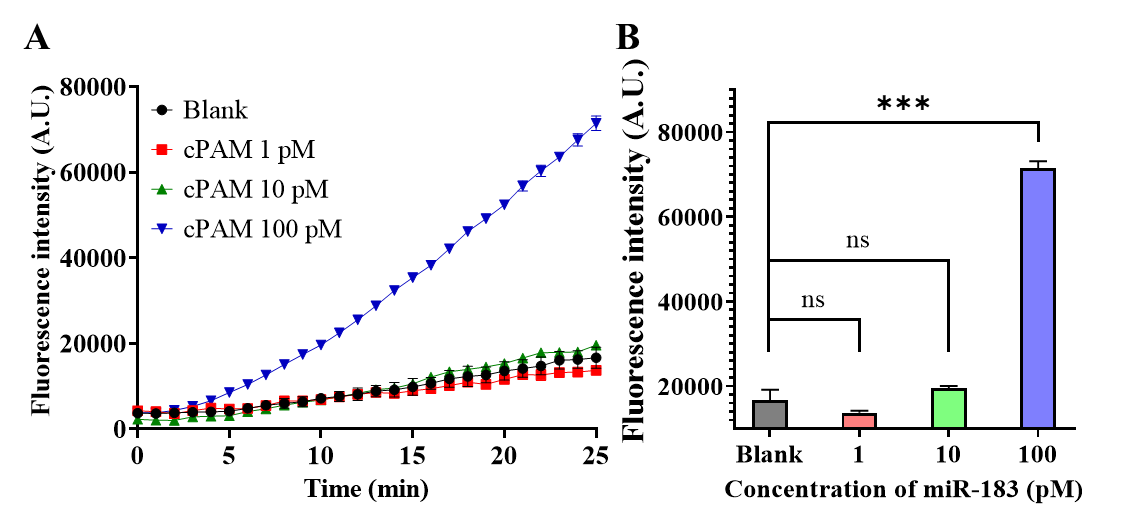


**Supplementary Figure 3.** cPAM-mediated miR-183 detection. A. Real-time fluorescence intensity of different concentrations. B. End-point fluorescence intensity of each group after a 25-minute detection. (Mean ± s.d. Variance was calculated using student's t-method, *** represents p<0.001.)

The cPAM-mediated miR-183 detection exhibited poor performance in detecting miR-183 at concentrations of lower than 10 pM.

# Abbreviated terms

CRISPR: Clustered regularly interspaced short palindromic repeats

sPAM: suboptimal protospacer adjacent motif

cPAM canonical protospacer adjacent motif

crRNA: CRISPR RNA

RCA: rolling-circle amplification

PT: padlock template

F-Q probe: fluorophore-quencher probe

T-ERCA: target triggered exponential rolling-circle amplification

SERS: surface-enhanced Raman spectroscopy

LAMP: loop-mediated amplification

SWV: square wave voltammetry

# References

Chen, P., Wang, L., Qin, P., Yin, B. C., & Ye, B. C. (2022). An RNA-based catalytic hairpin assembly circuit coupled with CRISPR-Cas12a for one-step detection of microRNAs. *Biosensors and Bioelectronics*, *207*(March), 114152. https://doi.org/10.1016/j.bios.2022.114152

He, Y., Yang, X., Yuan, R., & Chai, Y. (2017). “off” to “on” Surface-Enhanced Raman Spectroscopy Platform with Padlock Probe-Based Exponential Rolling Circle Amplification for Ultrasensitive Detection of MicroRNA 155. *Analytical Chemistry*, *89*(5), 2866–2872. https://doi.org/10.1021/acs.analchem.6b04082

Qing, M., Chen, S. L., Sun, Z., Fan, Y., Luo, H. Q., & Li, N. B. (2021). Universal and Programmable Rolling Circle Amplification-CRISPR/Cas12a-Mediated Immobilization-Free Electrochemical Biosensor. *Analytical Chemistry*, *93*(20), 7499–7507. https://doi.org/10.1021/acs.analchem.1c00805

Tang, X., Deng, R., Sun, Y., Ren, X., Zhou, M., & Li, J. (2018). Amplified Tandem Spinach-Based Aptamer Transcription Enables Low Background miRNA Detection. *Analytical Chemistry*, *90*(16), 10001–10008. https://doi.org/10.1021/acs.analchem.8b02471

Tian, W., Li, P., He, W., Liu, C., & Li, Z. (2019). Rolling circle extension-actuated loop-mediated isothermal amplification (RCA-LAMP) for ultrasensitive detection of microRNAs. *Biosensors and Bioelectronics*, *128*(October 2018), 17–22. https://doi.org/10.1016/j.bios.2018.12.041

Zhang, G., Zhang, L., Tong, J., Zhao, X., & Ren, J. (2020). CRISPR-Cas12a enhanced rolling circle amplification method for ultrasensitive miRNA detection. *Microchemical Journal*, *158*(July), 105239. https://doi.org/10.1016/j.microc.2020.105239

Zhou, S., Sun, H., Dong, J., Lu, P., Deng, L., Liu, Y., Yang, M., Huo, D., & Hou, C. (2023). Highly sensitive and facile microRNA detection based on target triggered exponential rolling-circle amplification coupling with CRISPR/Cas12a. *Analytica Chimica Acta*, *1265*, 341278. https://doi.org/10.1016/j.aca.2023.341278

# Figure Captions

**Supplementary Figure 1.** Comparison of 13 individual experiments of blank control with miR-183 at concentration of 1 aM under optimal conditions. (Mean ± s.d. Variance was calculated using student's t-method, *** represents p<0.001.)

**Supplementary Figure 2.** Detection of scramble ssDNA template by Cas12a. A. Real-time fluorescence of different concentration. B. Comparison of end-point fluorescence intensity for each group after a 60-minute detection. C. Comparison of end-point fluorescence intensity among cPAM, sPAM and nPAM at a concentration of 10 pM. (Mean ± s.d. Variance was calculated using student's t-method, * represents p<0.05, ** represents p<0.01.)

**Supplementary Figure 3.** cPAM-mediated miR-183 detection. A. Real-time fluorescence intensity of different concentrations. B. End-point fluorescence intensity of each group after a 25-minute detection. (Mean ± s.d. Variance was calculated using student's t-method, *** represents p<0.001.)
